# Bridging the Distance for Ischaemic Stroke Treatment in Regional Australia: A Retrospective Cohort Study

**DOI:** 10.1111/ajr.70177

**Published:** 2026-04-21

**Authors:** Stephanie Caldwell, Bronwyn Griffin, Ramon Navarro, Michael Crowe, Firas Alnidawi, Elizabeth Forster

**Affiliations:** ^1^ Townsville University Hospital Townsville Queensland Australia; ^2^ Griffith University Brisbane Queensland Australia; ^3^ Queensland Children's Hospital Brisbane Queensland Australia; ^4^ James Cook University Townsville Queensland Australia

**Keywords:** Australia, endovascular clot retrieval, regional, remote, risk factors, stroke, thrombolysis

## Abstract

**Background:**

One comprehensive stroke centre (CSC), in North Queensland, Australia, supports almost 700 000 people across 750 000 km^2^. Access to urgent ischaemic stroke treatment is challenging for remote and regional residents, who are more likely to carry stroke risk factors and have a 17% higher stroke incidence than those in major cities.

**Aims:**

To review and report on patient demographics, stroke characteristics, interventions, and outcomes of ischaemic stroke in this regional population.

**Methods:**

Retrospective review of admissions to a North Queensland CSC (March 2022—September 2023) with acute ischaemic stroke (ICD‐10‐AM: 163).

**Results:**

Among 305 patients, the median age was 72 years, 57% were male, and 10.5% identified as First Nations. Common stroke risk factors included hypertension (83%), smoking background (58%) and hypercholesterolaemia (57%). Stroke aetiology was unknown in 42%, followed by cardioembolic (31%). Median onset‐to‐emergency department time was 90 min, and to the CSC was 343 min. Thrombolysis was administered to 6% at the referring facility, and 7% on arrival to the CSC (median door‐to‐needle time, 73 min). Endovascular clot retrieval occurred in 11.7% of the primary presenters, with an overall successful recanalisation rate of 89%. The 6‐month mortality rate was 22%.

**Conclusions:**

High rates of modifiable risk factors require improved community management. Delayed presentation highlights potential gaps in stroke awareness and access to healthcare in this region. Reaching timely reperfusion targets should become a priority, which requires support from multiple stakeholders. The high proportion of unknown aetiologies may reflect incomplete stroke work‐up, documentation, or loss to follow‐up. Targeted stroke education for regional and remote communities and healthcare providers is recommended.

## Abstract (highlights)

**What is already known:**

- People living in regional Australia are more likely to suffer from stroke.
- Thrombolysis rates are lower in regional Australia and given in a less timely manner.
- There is one comprehensive stroke centre in Northern Queensland offering clot retrieval for a population of 700 000 and catchment area of around 750,000km^2^.

**What this paper adds:**

- Identified a high prevalence rate of modifiable stroke risk factors, highlighting the need for improved community‐level prevention and management.
- Describes stroke characteristics and management patterns before Telestroke service implementation.
- Demonstrates that acute reperfusion therapy rates remain lower than those in major cities.
- Highlighted the considerable delays in reaching the CSC, reducing the eligibility of reperfusion therapies.

## 1 Introduction

Regional Australians are 17% more likely to have a stroke than those in major cities [1]. In the Queensland state of Australia, 27% of residents live in regional or remote areas, and recent reports suggest a growing population [2]. Stroke risk factors such as hypertension, diabetes, smoking, obesity, and heart disease are more prevalent in regional and remote communities than in major cities [3]. Managing these risk factors, including atrial fibrillation and hyperlipidaemia, could significantly reduce stroke by up to 80% according to the Stroke Foundation [4].

If found to be a candidate, reperfusion therapies for ischaemic stroke patients can include thrombolysis and/or endovascular clot retrieval (ECR). Factors affecting eligibility include clinical presentation, time of symptom onset, medical history and imaging results [5]. Only 50% of outer regional health facilities offer 24‐h, 7‐day on‐site CT ability [6]. The lack of imaging availability to these areas may impact the number of candidates eligible for emergent reperfusion therapies. Thrombolysis rates in regional and remote Australia are less than those in urban populations [7] and remain below the national benchmark of 19% [8]. Efficient aeromedical retrieval enables excellent outcomes for remote large vessel occlusion stroke patients, despite the long distances to reach an ECR capable facility [9, 10].

Stroke patients managed in hospitals with no stroke units have poorer outcomes [11]. To combat the disadvantages within regional and remote Australia, Telestroke programmes have been implemented to connect hospitals to specialists enabling prompt assessment and recommendations [12, 13, 14]. Townsville University Hospital (TUH) is the only comprehensive stroke centre in Northern Queensland, offering both intravenous thrombolysis and ECR. As an outer regional facility, TUH provides specialist stroke services to a catchment population of approximately 700,000 individuals, while covering a geographical area of over 750 000 km^2^ that extends from Mackay to the Torres Strait Islands and westward to the Northern Territory border [15, 16].

### 1.1 Aims

This study aimed to review and report patient demographics, stroke characteristics, management, treatment and outcomes of ischaemic stroke for presentations to TUH. Data on risk factors were collected to identify where a focus on preventative stroke health is required. Identifying geographical locations and transfer times was crucial for potentially identifying areas for improvement and increasing the number of candidates eligible for reperfusion therapies due to time constraints.

## 2 Methods

### 2.1 Study Design

This retrospective observational study was conducted at a regional comprehensive stroke centre (CSC) in North Queensland, Australia. Patients were eligible for inclusion if they were aged 18 years or over, admitted between March 2022 and September 2023, and had a confirmed diagnosis of ischaemic stroke (ICD‐10‐AM:163). The study period was selected to align with the introduction of endovascular clot retrieval (ECR) at the centre, with an 18‐month timeframe considered sufficient to obtain an adequate sample size and capture the early experiences.

Patients were excluded if admission was solely for post‐stroke rehabilitation, for a post‐stroke procedure (e.g., carotid endarterectomy) without an acute stroke admission, or if subsequent imaging or clinical review excluded an ischaemic stroke diagnosis. Potential cases were identified by the hospital data analytics team using the ICD coding. This study was conducted and reported in accordance with the Strengthening and Reporting of Observational Studies in Epidemiology (STROBE) guidelines for cohort studies (File S1).

### 2.2 Data Extraction

Data extracted from the electronic medical record included:

- Demographics: age, sex, post‐code, First Nations status, body mass index, pre‐morbid modified Rankin Scale (mRS) score, National Institutes of Health Stroke Scale (NIHSS) score on arrival.
- Stroke risk factors: hypertension, atrial fibrillation, hyperlipidaemia, diabetes, prior stroke or transient ischaemic attack, smoking, cardiovascular disease. Known history was determined as yes if medicated for that risk factor before admission or documented known history on admission.
- Stroke aetiology: cardioembolic, atherosclerosis, lacunar, other or unknown (based on the Trial of ORG10172 in Acute Stroke Treatment (TOAST)) criteria [17].
- Intervention: thrombolysis, ECR, stroke prevention medication prescription.
- Outcome: mortality, length of stay, discharge destination, NIHSS score on Day 1 and on discharge, and post‐morbid mRS score.

Postcodes were categorised according to the Australian Statistical Geography Standard (ASGS), which uses five classes to define remoteness: major cities, inner regional, outer regional, remote and very remote [18]. An mRS score of 0 indicates that the person had no symptoms and a score of 2 indicates slight disability though able to look after their own affairs.

### 2.3 Statistical Analysis

Descriptive analysis was used to summarise the data. Categorical variables were presented as frequencies and percentages. For the continuous variables, median and interquartile ranges (IQR) were chosen. Kruskal–Wallis, Pearson, and Wilcoxon tests were used to describe the differences between groups for categorical and continuous variables, as appropriate. The analysis was completed in Jamovi (version 2.6.44) using the ClinicoPathDescriptives module (version 0.0.2) for crosstabs. A *p*‐value of < 0.05 was considered statistically significant. Due to the retrospective nature of the study, missing data occurred for some variables, particularly functional outcome measures, which were not routinely documented. Analyses were conducted using available‐case data, with the extent of missingness reported in the tables.

### 2.4 Ethical Considerations

Ethics approval was obtained by the Townsville Hospital and Health Service Audit, Quality and Innovation Review (AQUIRE) Panel (Reference number: THHSAQUIRE1660) and Griffith University Human Research Ethics Committee (GU number: 2024/124). A waiver of consent was granted for this retrospective analysis of deidentified data from patient records.

## 3 Results

A total of 323 patients were identified by the hospital's data analytics team using the ICD‐10‐AM code of 163. Eighteen patients were excluded; 10 had no confirmed ischaemic stroke following initial investigation (e.g., follow‐up imaging/consultation), and five were admitted solely for a carotid endarterectomy procedure. This left a total of 305 eligible patients included in the analysis. An additional seven patients experienced a recurrent stroke during the study period; however, only data from their initial stroke presentation were analysed.

### 3.1 Patient Characteristics

Of the 305 patients, the majority of stroke patients were male (57%, *n* = 173), and the median age was 72 years [IQR 60, 80]. As per the TOAST criteria, unknown was the most common stroke cause with 42%. First Nations patients accounted for 10.5% of the cohort and were significantly younger than non‐Indigenous patients, with median ages of 51 and 74 years, respectively (*p* < 0.01). Characteristics of the patients are presented in Table 1.

**TABLE 1:** Patient characteristics (*n* = 305).

Characteristic	Frequency (%)/Median [IQR]
Age, years	72 [60, 80]
Sex, male	173 (57%)
*First Nations peoples*	32 (10.5%)
Aboriginal	23 (8%)
Both Aboriginal & Torres Strait Islander	8 (3%)
Torres Strait Islander	1 (0.3%)
*Geographic location*	
Major city	3 (1%)
Inner regional	9 (3%)
Outer regional	264 (87%)
Remote	11 (4%)
Very remote	9 (3%)
Interstate	7 (2%)
Overseas	2 (1%)
*Stroke risk factors*	
Atrial fibrillation (AF) (*n* = 303)	100 (33%)
Known AF	57 (19%)
Known AF anticoagulated prior	21 (37%)
Hypertension	253 (83%)
Known hypertension	213 (70%)
Known hypertension on antihypertensives prior	186 (87%)
Diabetes mellitus (*n* = 302)	114 (38%)
Known diabetes	104 (34%)
HbA1c, mmol/L (*n* = 281)	5.8 [5.4, 7.1]
HbA1c (diabetic only), mmol/L	7.5 [6.7, 9.3]
Previous stroke/TIA	63 (21%)
LDL ≥ 2.5 mmol/L (*n* = 272)	154 (57%)
Known hyperlipidaemia	148 (49%)
Smoking (ever/current) (*n* = 231)	135 (58%)
Cardiovascular disease (HF, IHD, RHD)	106 (35%)
BMI kg/m^2^ (*n* = 279)	28 [24, 32]
*Pre‐morbid status (n = 235)*	
Pre‐morbid mRS 0–2	195 (83%)
Pre‐morbid mRS 3–5	40 (17%)
NIHSS on arrival (*n* = 218)	6 [2, 12]

*Note:* Data presented as frequency (%) or median [IQR].

Abbreviations: AF, atrial fibrillation; BMI, body mass index; HbA1c, haemoglobin A1C; HF, heart failure; IHD, ischaemic heart disease; LDL, low‐density lipoprotein; mRS, modified Rankin Scale; NIHSS, National Institute of Health Stroke Scale; RHD, rheumatic heart disease.

### 3.2 Risk Factors

Atrial fibrillation (AF) was newly identified in 14% of patients (*n* = 43), and 19% (*n* = 57) of the cohort had a known history of AF (Table 1). In the group with known AF, only 37% (*n* = 21) of them were anticoagulated in the community prior, and a further 7% of the total cohort were anticoagulated for other reasons. Reasons for not anticoagulating those with AF in the community were found in 16 cases (File S2).

Hypertension was the most common risk factor (83%); of these, 84% were aware of their diagnosis before admission, and 87% were prescribed antihypertensive medication. Patient with diabetes mellitus made up 38%, with 9% having a new diagnosis during their admission. Hyperlipidaemia was reported in 49% of patients, yet 57% had elevated lipid levels (LDL ≥ 2.5 mmol/L) on admission. All other risk factors are described in Table 1.

### 3.3 Stoke Interventions

Thrombolysis was administered to 6% of patients at their referring facility, and the CSC thrombolysed a further 7% upon arrival. The median door‐to‐needle time was 73 min [IQR 65, 114]. Endovascular clot retrieval was performed in 11.7% for those who presented directly to the CSC. The median door‐to‐groin time was 105 min [IQR 75, 138] in primary presenters, and 85 min [IQR 49, 131] in the IHT group, though not statistically significant (Wilcoxon F (1.55) = 2.87, *p* = 0.10). The overall median ictus to recanalisation time was 147 min [IQR 111, 204]. Successful recanalisation (TICI 2b‐3) was achieved in 89% of ECR cases. Two procedures were aborted: one due to the inability to gain access, and the other due to the discovery of a sinus venous thrombosis. Secondary stroke preventative medication prescribing was found to be sufficient (File S2).

### 3.4 Patient Journey

**FIGURE 1:**
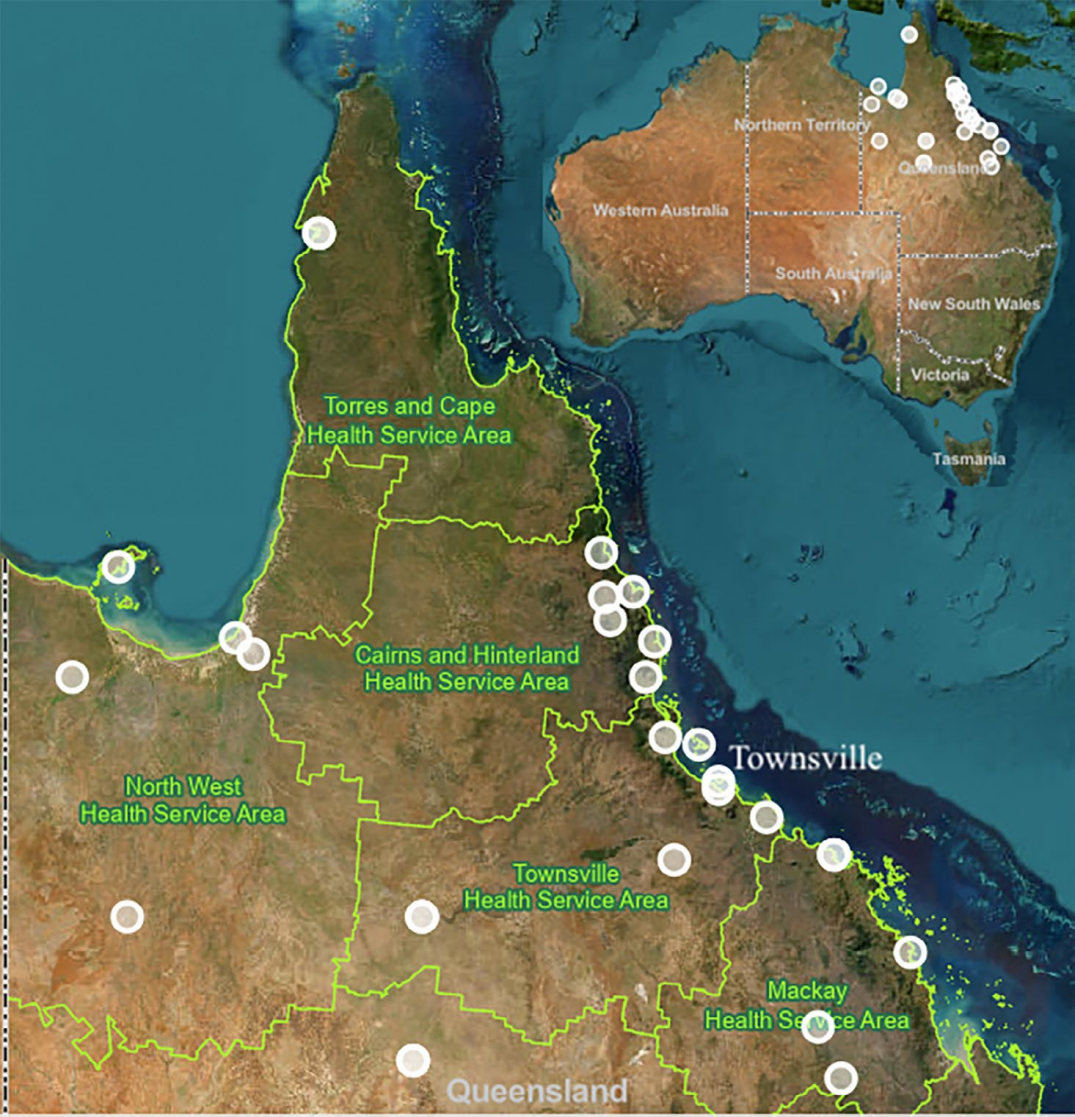
Interhospital transfer locations from North Queensland, Australia and the health service boundaries [16]. Furthest distance by air: Weipa, 892 km; Mornington Island, 853 km; Doomadgee, 850 km; Mount Isa, 777 km.

Patients presenting directly to the CSC had a more prolonged median onset to ED presentation time of 151 min [IQR 68, 744] compared with those who transferred from another hospital (90 min [IQR 49–174]). The time taken for the onset of symptoms to transfer to the CSC was a median of 401 min [IQR 300, 695]. A total of 109 patients (36%) were transferred to the CSC from another facility by either road or air (Figure 1). For patients with a single transfer, the median door‐in‐door‐out (DIDO) time was 189 min [IQR 134, 311]. Nine of those patients (8%) required more than one transfer stop.

### 3.5 Patient Outcomes

Overall, 40% of patients were discharged home, 28% were discharged to inpatient rehabilitation, 13% were transferred to another hospital, 5% returned to their previous residential aged care facility (RACF), 2% were discharged to a new RACF, and 12% of patients died in hospital. The 6‐month mortality rate was 22%. The median length of stay (LOS) for acute care was 6 days, and for those in the rehabilitation phase, LOS was 41 days (Tables 2 and 3).

**TABLE 2:** Stroke interventions.

Stroke interventions	Frequency (%)/Median [IQR]	National level (%)/Median	Target
Code stroke	179 (58%)	—	—
*Thrombolysis*			
Thrombolysis at CSC (primary presenters, *n* = 196)	22 (7%)	(7%) outer regional^a^ (11%) regional^b^	(19%)^b^
Thrombolysis at referring facility prior to CSC (IHT cohort, *n* = 109)	19 (6%)		
Thrombolysis total cohort (*n* = 305)	41 (13%)	—	—
Door‐to‐needle time of primary presenters, min (*n* = 22)	73 [65, 114]	80^a^–74^b^	< 60^b^
*Endovascular clot retrieval (ECR)*			
ECR (primary presenters, *n* = 196)	23 (11.7%)	(15%)^b^	(23%)^b^
ECR (IHT cohort, *n* = 109)	34 (31.2%)		
ECR overall	57 (19%)	—	—
Door‐to‐groin time of IHT cohort, min (*n* = 34)	85 [49, 131]		< 30 ^b^ < 90^b^
Door‐to‐groin time of primary presenters, min (*n* = 23)	105 [75, 138]	115^b^	> 90^b^
Door to recanalisation time, min (*n* = 50)	147 [111, 204]	134 [89, 184]^b^	—
TICI score 2b‐3 (*n* = 55)	49 (89%)	—	—

*Note:* Data presented as frequency (%) or median [IQR].

^a^ Stroke Foundation, National stroke audit 2023 [19].

^b^ AusCr 2022 report [8].

**TABLE 3:** Patient journey details.

	Frequency (%)/Median [IQR]
*Inter‐hospital transfer cohort (n = 109)*	
IHT	109 (36%)
1 hospital stop	100 (92%)
2 hospital stops	9 (8%)
Ictus‐to‐first ED time, min (*n* = 88)	90 [49, 174]
DIDO time, min (*n* = 104)	189 [134, 322]
DIDO at second hospital time, min (*n* = 9)	1668 [367, 2358]
Ictus‐to‐CSC time (IHT cohort), min (*n* = 93)	401 [296, 697]
*Non‐IHT cohort (n = 196)*	
Ictus‐to‐ED time, minutes (*n* = 188)	151 [68, 744]

*Note:* Data presented as frequency (%) or median [IQR].

Abbreviations: CSC, comprehensive stroke centre; DIDO, door‐in‐door‐out; ED, emergency department; IHT, interhospital transfer.

## 4 Discussion

This study highlights the complex realities of stroke presentations across the geographically dispersed region of North Queensland. The insights gained align with, but also add depth to, previous work examining stroke care in regional and remote Australian contexts.

### 4.1 Patient and Stroke Characteristics

Consistent with national trends [20], the majority of stroke patients were male. Strikingly, First Nations patients were significantly younger, with a 20‐year median age gap. This reflects persistent health inequities and aligns with national data showing a younger age of stroke onset in Indigenous Australians [21]. Furthermore, over one‐third of all patients were under the age of 65, reinforcing the burden of stroke in younger populations and the need for tailored prevention strategies.

The high proportion of strokes with unknown aetiology warrants further investigation, as this may reflect incomplete diagnostic work‐up, insufficient documentation, or loss to follow‐up. Introducing a standardised stroke proforma could provide a practical approach to improve diagnostic consistency and quality of care.

### 4.2 Opportunities for Prevention

More than 80% of strokes can be prevented [22], highlighting the importance of timely diagnosis and adequate management of modifiable risk factors. The Australian National Preventive Health Strategy 2021–2030 [23] recognises that reducing chronic diseases in remote populations requires coordinated, multi‐layered interventions across the life course to address risk factors that are shaped by social, environmental, and health‐system influences.

There was a high prevalence of hypertension, diabetes mellitus, dyslipidaemia and atrial fibrillation, slightly higher than some other regional studies [12, 24, 25]. Rates were, however similar in another regional Queensland city, Toowoomba [26]. Hypertension was identified as the greatest stroke risk factor, with many patients on at least one antihypertensive agent. As a GP can be difficult to access for regional and remote populations, initiatives such as expanding practice for rural community pharmacists to include prescribing can support rural GPs by reducing care burden and enhancing timely access to medicines and care for consumers in these communities [27].

Untreated AF was noted in over half the patients, raising concerns about undertreatment, and missed opportunities for stroke prevention since a third of ischaemic strokes were cardioembolic. This is supported by the finding in a regional Australian study, where untreated AF was the most common (75%) cause of cardioembolic stroke [26]. National guidelines suggest oral anticoagulation therapy to prevent stroke in patients with non‐valvular AF whose CHA2DS2‐VA score is 2 or more, unless there are contraindications to anticoagulation [28]. Not anticoagulating some patients is reasonable, as risks can outweigh the benefits in some cases, which was noted in this study. Opportunistic screening in the community should be conducted in those aged over 65 years [28]. Strengthening primary prevention in the community setting with targeted strategies is necessary to identify and manage these risk factors.

Smoking history was high, though the study did not differentiate between current or historical, and generally, smoking rates are higher in remote communities [29]. The median BMI was reported to be in the overweight range, further underscoring the burden of modifiable risk factors. Due to the barriers in accessing primary healthcare in regional and remote areas, strategies such as online resources, community education and awareness, telehealth, remote medical training and staff retention are considered essential steps to address these modifiable risk factors [30].

First Nations people were identified within this study as an important priority population for stroke prevention and care, consistent with previous research [21, 31, 32]. Culturally responsive strategies that are codesigned and led in partnership with Aboriginal and Torres Strait Islander communities are essential to address inequities in stroke and risk outcomes [21]. Aboriginal and Torres Strait Islander health workers, when supported with knowledge and resources, are well placed to lead community awareness campaigns and influence prevention [33]. Government‐backed programmes such as the ‘Deadly Choices campaign’ [34] promote healthy lifestyles and encourage annual Aboriginal and Torres Strait Islander health assessments (‘715 Health Checks’). Local Aboriginal Community Controlled Health Services, such as the Townsville Aboriginal and Islander Health Service (TAIRS), use these checks to identify stroke risk factors like hypertension, diabetes, cardiac arrhythmias and abnormal blood results [19]. These trusted, culturally safe services provide ideal opportunities to embed targeted stroke education and reinforce the importance of seeking urgent care for stroke symptoms.

### 4.3 Access and Timeliness of Care

The vast geography of North Queensland presents substantial challenges for timely stroke care, with many communities located hundreds of kilometres from the single CSC and reliant on aeromedical retrieval. The region is comparable in size to Texas and California combined, or the entire state of New South Wales in Australia, highlighting the scale of the logistical challenges in delivering equitable acute stroke care.

National Stroke Targets (30/60/90) were designed to benchmark high‐quality acute stroke care [20]. In this study, door‐to‐needle time was comparable to other regional settings [8, 35]; however, opportunities remain to further reduce treatment delays and reach the national performance targets. While CT perfusion for extended‐window therapies has been recommended, availability across remote centres is still evolving across the region [36].

Interpretation of ECR rates requires careful interpretation. Although 19% of ischaemic stroke patients admitted to the CSC underwent the procedure, this figure does not represent the true regional rate, as many patients never present to the CSC. Among primary presenters, the ECR rate was similar to the national regional average (12%), although it was below the benchmark of 23% achieved by top‐performing metropolitan centres [8]. Considering this reflects the initial stages when ECR was implemented in the region, it has been a great achievement. Median door‐to‐groin times aligned with national figures [20], although exceeded the optimal target recommended by the Stroke Foundation [35]. As highlighted by Manzoor et al. [10], improving performance will require ongoing investment in workforce training, enhanced pre‐hospital triage, and streamlined IHT processes to reduce treatment delays and expand equitable access to stroke interventions in regional Queensland.

Patient journey time metrics further reveal that prolonged door‐in‐door‐out (DIDO) times at referring hospitals significantly contributed to treatment delays, particularly when multiple transfer legs were required. Although no national DIDO time target exists for aeromedical retrievals, delays heighten the risk of missing eligibility windows for reperfusion access. The Royal Flying Doctors Service (RFDS) plays a critical role in connecting remote patients to stroke centres, yet barriers persist. One national study estimated the median flight distance with the RFDS was 290 km, with a median transfer time of 4 h for stroke patients [37].

These findings align with the work of Tiu et al. [38], who identified pre‐hospital delays and prolonged DIDO times as major barriers to regional reperfusion access, recommending aggressive monitoring of DIDO performances and streamlined transfer protocols. Numerous factors may impact timely presentation, ranging from geographical barriers, low education levels, delayed recognition of stroke and its urgency and overall hesitation to seek help [31, 39]. Telestroke programmes have demonstrated improvements in treatment metrics and mortality in regional settings [13]. In Queensland, the phased rollout of the Telestroke Service represents a promising avenue to accelerate treatment decisions, reduce DIDO times, and optimise the limited therapeutic window for reperfusion therapies.

### 4.4 Stroke Outcomes

Despite the barriers to access the ECR‐capable facility, outcomes were encouraging, with 89% of patients achieving successful recanalisation (TICI 2b‐3). This supports the effectiveness of a centralised stroke ECR service when patients can access it in time, as other studies have also found [9, 10, 36]. Majority of patients were discharged on secondary prevention medications, suggesting good adherence to guideline‐based care in the subacute phase.

In‐hospital mortality was 12%, with a 6‐month mortality of 22%, aligning with previous reports from regional and remote stroke studies [26, 32, 40]. Functional outcome measurement was limited due to missing documentation of discharge NIHSS and post‐stroke mRS, which limits comparisons and quality improvement benchmarking. However, a recent study in the same demographic and location found that 47% of stroke patients post ECR had a 90‐day mRS score of 0–2 [10], supporting the critical need for this regional service. Changes were implemented soon after data completion to allow for improved stroke documentation. Introducing a standardised electronic stroke proforma could provide improved diagnostic documentation and quality of care.

## 5 Limitations

Due to the retrospective nature of this study, multiple data points were missing from patient medical records. Missingness primarily reflected variability in routine clinical documentation rather than study design. Remoteness classification was determined using the listed postcode; however, this may not reflect where they were at the time of stroke onset. Stroke aetiology was not always clearly stated in the physician notes; therefore, aetiology was assumed from the workup results, risk factors, and location of stroke. The study did not identify if risk factors were managed effectively, only that they existed by diagnosis in the notes or if medication was prescribed to manage the specific diagnosis. For example, patients prescribed antihypertensive or lipid‐lowering medications were assumed to have a diagnosis of hypertension or hyperlipidaemia, respectively.

A small proportion (8.5%) of stroke aetiologies were flagged for review by a Neurologist to confirm the aetiology. Stroke workup was not complete in all cases, resulting in unknown cause of stroke. This study included only patients who presented to the regional CSC; many patients are not transferred for a range of reasons, including patient preference, medical unsuitability, transport delays, or deemed an unlikely candidate for reperfusion treatment. The LOS may not be reflective of the true time spent within an acute care setting, as some patients were palliated or awaited nursing home placement. In addition, the onset time of symptoms or interhospital transfer times were missing or inaccurate in some cases and were therefore excluded from the time‐based analyses. There was a lack of documentation to effectively measure the stroke outcomes in relation to day 1 NIHSS, discharge NIHSS, post‐morbid mRS as this is typically only reported post reperfusion patients.

## 6 Conclusions

This study reinforces the unique demographic and geographic challenges of stroke care in regional and remote North Queensland. The younger age at stroke among First Nations people, high rates of modifiable stroke risk factors and delayed hospital presentation should be key targets for change and future research. Policy and service development, including the implementation of Telestroke, requires support from multiple stakeholders, which is crucial for increasing the number of patients eligible for reperfusion and meeting time targets. Innovative strategies to educate the community on stroke awareness and reduce DIDO time may help close the gap in acute stroke access for regional populations.

## Author Contributions

**Stephanie Caldwell:** conceptualization, methodology, investigation, formal analysis, writing – review and editing, writing – original draft, validation. **Bronwyn Griffin:** writing – review and editing, visualization, methodology, supervision. **Ramon Navarro:** visualization, methodology, writing – review and editing, supervision, validation. **Michael Crowe:** formal analysis, writing – review and editing, methodology, data curation, validation. **Firas Alnidawi:** validation, writing – review and editing. **Elizabeth Forster:** writing – review and editing, visualization, supervision, methodology.

## Funding

This work was supported by SERTA Research Seed Fellowship (Grant THHSSERTA_RSFG1_2024).

## Ethics Statement

Ethics approval was obtained by the Townsville Hospital and Health Service Audit, Quality and Innovation Review (AQUIRE) Panel (Reference number: THHSAQUIRE1660) and Griffith University Human Research Ethics Committee (GU Ref No: 2024/124). A waiver of consent was granted for this retrospective analysis of deidentified data from patient records.

## Conflicts of Interest

The authors declare no conflicts of interest.

## Supporting information


**File S1:** STROBE Statement—checklist of items that should be included in reports of observational studies.

**File S2:** ajr70177‐sup‐0002‐FileS2.docx.
**Table S1A**. Reasons for not anticoagulating in the community with known atrial fibrillation.
**Table S2B**. Discharge prescriptions for stroke preventative medications.

## Data Availability

The datasets used and/or analysed during the current study are available from the corresponding author on reasonable request.
