# Supplementary material for: Bridging the Distance for Ischaemic Stroke Treatment in Regional Australia: A Retrospective Cohort Study
Source: Aust J Rural Health. 2026 Apr 21;34(2):e70177. doi: 10.1111/ajr.70177 (PMC13097238; doi:10.1111/ajr.70177)
Supplement: Supplementary file 2 — File S2: ajr70177‐sup‐0002‐FileS2.docx. Table S1A. Reasons for not anticoagulating in the community with known atrial fibrillation. Table S2B. Discharge prescriptions for stroke preventative medications. [file AJR-34-0-s002.docx]

**Supplementary file 2.**

Table A. Reasons for not anticoagulating in the community with known atrial fibrillation

| Reason for not anticoagulating | Frequency  N=16 |
| --- | --- |
| Bleeding | 5 |
| Falls risk | 2 |
| Ran out of prescription | 1 |
| Recent surgery | 5 |
| Intolerance | 1 |
| Non-compliant | 2 |

Table B. Discharge prescriptions for stroke preventative medications

| Discharge prescriptions | *Frequency (%) / Median [IQR]* | National level | Target |
| --- | --- | --- | --- |
| Antihypertensives | 185 (71%) | (82%)^^*^ | (94%) ^+^ |
| Lipid-lowering | 242 (93%) | (94%)^^*^ | (97%) ^+^ |
| Antithrombotic | 256 (98%) | (99%)^^*^ | (99%) ^+^ |

+ AusCr 2022 report ^18^

^ Stroke Foundation, National stroke audit 2023 ^19^

* Excludes those contraindicated, futile, refused and only included those discharged to community
